# Should Patients Suffering from Degenerative Cerebellar Ataxia Switch from Balance Training to Aerobic Exercise?

**DOI:** 10.1002/mdc3.70427

**Published:** 2025-11-06

**Authors:** Winfried Ilg, Sarah Milne

**Affiliations:** ^1^ Section Computational Sensomotorics Hertie Institute for Clinical Brain Research Tübingen Germany; ^2^ Centre for Integrative Neuroscience (CIN) Tübingen Germany; ^3^ Bruce Lefroy Centre for Genetic Health Research, Murdoch Children's Research Institute Parkville Victoria Australia; ^4^ Department of Paediatrics Melbourne University Melbourne Victoria Australia; ^5^ Physiotherapy Department, Monash Health Clayton Victoria Australia; ^6^ School of Physiotherapy Monash University Frankston Victoria Australia

**Keywords:** degenerative cerebellar ataxia, neurorehabilitation

After long‐standing doubts about whether motor training has any relevant positive effects in patients with degenerative cerebellar ataxia, increasing evidence suggests that continuous, intensive exercise can reduce ataxia symptoms and improve everyday mobility. Most of these programs focus on balance and coordination training,^1^, ^2^ sometimes combined with exercises for strengthening and functional mobility.^3^ While these motor improvements have been confirmed in various studies, including home training,^2^ there are still questions regarding the optimal rehabilitation program depending on the severity of ataxia and the optimal dosage, as well as whether training can achieve disease‐modifying effects in addition to functional improvement.

Barbuto and colleagues^4^ recently conducted a controlled trial examining aerobic exercise as a rehabilitation strategy for improving ataxia. They compared the effects of home‐based high‐intensity aerobic cycling training and balance training on symptoms of cerebellar ataxia over 12 months. Training was performed for 30 minutes, five times per week, with aerobic participants exercising at up to 85% predicted maximum heart rate, while balance participants completed progressively challenging balance exercises. The primary outcome was ataxia severity measured by the Scale for the Assessment and Rating of Ataxia (SARA), with secondary outcomes including fatigue, aerobic capacity, gait, and balance. Aerobic training resulted in significantly greater improvements than balance training in SARA scores. According to the authors, these results suggest that aerobic training is more effective than balance training at improving symptoms of ataxia, reducing fatigue, and increasing overall fitness.

We fully agree that a decline in fitness and early fatigue can exacerbate the symptoms of ataxia, and that aerobic training is therefore a beneficial part of an exercise program, especially in the advanced stages of the condition when other types of fitness training are no longer possible. Furthermore, mouse studies have demonstrated the neuroprotective effects of endurance training on the progression of neurodegeneration.

However, we are concerned that the conclusion of the authors of this study: “high‐intensity aerobic training may offer greater functional benefits than balance training,” may lead to balance exercises no longer being recommended for people with ataxia. Given the consistent evidence demonstrating the effectiveness of balance training,^5^ we consider removing balance exercises from the repertoire of rehabilitation interventions will lead to detrimental outcomes for people with ataxia. The cerebellum is essential for coordinating movement and controlling balance, so these abilities, which are directly affected by the disease, must be specifically trained to prevent premature loss of functional mobility.

Barbuto and colleague's^4^ findings of lower improvements in the balance groups also highlight the necessity and difficulty of finding the appropriate level of challenge for balance exercises tailored to the individual severity of symptoms, particularly in the home environment. The authors have divided the exercises into three groups (easy, moderate, difficult). For the easy exercises, participants are allowed to hold on with both hands, and for the moderate exercises with one hand. However, this strategy, which is understandable for safety reasons, means that participants who are moderately to severely affected do not train their balance because they hold on to something during training.

An earlier home balance exercise study^2^ has shown that improvements in walking are significantly associated with appropriate challenges of balance exercises.

In summary, we would like to emphasize the importance of a mix of exercise strategies, including aerobic, strengthening, coordination, and balance training. Further research is needed to optimize individualized rehabilitation depending on disease stage. In the interests of individuals with cerebellar ataxia, we would strongly advise against reducing ataxia rehabilitation to aerobic exercise, as this article suggests.

## Author Roles

(1) Research project: A. Conception, B. Organization, C. Execution; (2). Statistical Analysis: A. Design, B. Execution, C. Review and Critique; (3). Manuscript Preparation: A. Writing of the first draft, B. Review and Critique; In this “In Focus” contribution, there is no own research and statistical analysis.

W.I.: 3A, 3B.

S.M.: 3A, 3B.

## Disclosures

**Ethical Compliance Statement:** Ethical approval is not applicable here, as our contribution is a commentary on another published study with different authors. Informed patient consent was not necessary for this work. We confirm that we have read the Journal's position on issues involved in ethical publication and affirm that this work is consistent with those guidelines.

**Funding Sources and Conflict of Interest:** No specific funding was received for this work. The authors declare that there are no conflicts of interest relevant to this work.

**Financial Disclosures for the previous 12 months:** The authors declare that there are no additional disclosures to report.

## Data Availability

Data sharing not applicable to this article as no datasets were generated or analysed during the current study.

## References

[1] Ilg W , Synofzik M , Brotz D , Burkard S , Giese MA , Schols L . Intensive coordinative training improves motor performance in degenerative cerebellar disease. Neurology 2009;73(22):1823–1830. 19864636 10.1212/WNL.0b013e3181c33adf

[2] Keller JL , Bastian AJ . A home balance exercise program improves walking in people with cerebellar ataxia. Neurorehabil Neural Repair 2014;28(8):770–778. 24526707 10.1177/1545968314522350 PMC4133325

[3] Milne SC , Roberts M , Williams S , et al. Goal‐directed rehabilitation versus standard Care for Individuals with hereditary cerebellar ataxia: a multicenter, single‐blind, randomized controlled superiority trial. Ann Neurol 2025;97(3):409–424. 39520242 10.1002/ana.27130

[4] Barbuto S , Lee S , Stein J , Kuo SH , Quinn L , Spinner M , Stern Y . Home training for cerebellar ataxias: a randomized clinical trial. JAMA Neurol 2025. 10.1001/jamaneurol.2025.3421. PMC12434599 40946705

[5] He M , Zhang HN , Tang ZC , Gao SG . Balance and coordination training for patients with genetic degenerative ataxia: a systematic review. J Neurol 2021;268(10):3690–3705. 32583055 10.1007/s00415-020-09938-6

